# Age-Related Mitochondrial DNA Depletion and the Impact on Pancreatic Beta Cell Function

**DOI:** 10.1371/journal.pone.0115433

**Published:** 2014-12-22

**Authors:** Donna L. Nile, Audrey E. Brown, Meutia A. Kumaheri, Helen R. Blair, Alison Heggie, Satomi Miwa, Lynsey M. Cree, Brendan Payne, Patrick F. Chinnery, Louise Brown, David A. Gunn, Mark Walker

**Affiliations:** 1 Institute of Cellular Medicine, Newcastle University, Newcastle upon Tyne, NE2 4HH, United Kingdom; 2 Newcastle University Institute for Ageing, Newcastle University, Newcastle upon Tyne, NE4 5PL, United Kingdom; 3 Department of Obstetrics and Gynaecology, Faculty of Medical and Health Sciences, University of Auckland, Auckland 1142, New Zealand; 4 Wellcome Centre for Mitochondrial Research, Institute of Genetic Medicine, Newcastle University, Newcastle upon Tyne, NE1 3BZ, United Kingdom; 5 Unilever R&D, Colworth Science Park, Sharnbrook, Bedford, MK44 1LQ, United Kingdom; RIKEN Advanced Science Institute, Japan

## Abstract

Type 2 diabetes is characterised by an age-related decline in insulin secretion. We previously identified a 50% age-related decline in mitochondrial DNA (mtDNA) copy number in isolated human islets. The purpose of this study was to mimic this degree of mtDNA depletion in MIN6 cells to determine whether there is a direct impact on insulin secretion. Transcriptional silencing of mitochondrial transcription factor A, *TFAM*, decreased mtDNA levels by 40% in MIN6 cells. This level of mtDNA depletion significantly decreased mtDNA gene transcription and translation, resulting in reduced mitochondrial respiratory capacity and ATP production. Glucose-stimulated insulin secretion was impaired following partial mtDNA depletion, but was normalised following treatment with glibenclamide. This confirms that the deficit in the insulin secretory pathway precedes K^+^ channel closure, indicating that the impact of mtDNA depletion is at the level of mitochondrial respiration. In conclusion, partial mtDNA depletion to a degree comparable to that seen in aged human islets impaired mitochondrial function and directly decreased insulin secretion. Using our model of partial mtDNA depletion following targeted gene silencing of *TFAM*, we have managed to mimic the degree of mtDNA depletion observed in aged human islets, and have shown how this correlates with impaired insulin secretion. We therefore predict that the age-related mtDNA depletion in human islets is not simply a biomarker of the aging process, but will contribute to the age-related risk of type 2 diabetes.

## Introduction

The prevalence of type 2 diabetes has been found to increase with advancing age [1]–[3]. This is thought to be due in part to the age-related progressive decline in pancreatic beta cell function [4], resulting in an age-related decrease in insulin secretion [5] and abnormal glucose tolerance [6]. But it is still unknown what mechanisms contribute to this age-related decline in pancreatic beta cell function.

Mitochondrial DNA (mtDNA) is a circular double stranded DNA molecule of 16.6 kb in length in humans [7] and encodes 13 polypeptides essential for mitochondrial oxidative phosphorylation [8], [9]. Insulin secretion is heavily dependent upon the ATP produced following glucose metabolism and mitochondrial oxidative phosphorylation, and occurs following ATP-gated K^+^ channel closure, subsequent membrane depolarisation and Ca^2+^-stimulated insulin exocytosis [8], [10]. Inherited mutations in the mitochondrial genome have been estimated to account for approximately 1% of all diabetes cases [11]. It is known that certain mtDNA abnormalities are strongly associated with diabetes, particularly the A3243G mutation, which has been shown to result in impaired insulin secretion [12], [13].

An age-related decline in mtDNA copy number has been reported in numerous human tissues [14]–[17]. In human pancreatic islets, it has been shown that mtDNA copy number was significantly reduced in non-diabetic islet donors aged ≥50 years compared to donors aged ≤50 years; and that the mean mtDNA copy number decreased by 50% in individuals aged between 17 and 75 years [14]. The study assessed mtDNA copy number in hand-picked whole islets, obtained from 15 non-diabetic pancreas donors; there was a significant negative correlation between mtDNA copy number and advancing age. Although the 50% reduction in mtDNA copy number reported by Cree *et al.* was from whole islets and was not exclusively beta cells, it has been well documented *in vitro* that severe (>90%) mtDNA depletion in insulin secreting beta cell lines results in decreased insulin secretion [18]–[21]. Mutations of the thymidine kinase 2 (TK2) gene result in mtDNA depletion in skeletal muscle, but cytochrome *c* oxidase (COX) deficiency as a marker of mitochondrial dysfunction was only seen in the presence of severe (≥95%) mtDNA depletion [22]. So the question arises as to whether the 50% mtDNA depletion observed in aged human islets is sufficient to impair mitochondrial function and insulin secretion, or whether it is functionally well tolerated as seen in skeletal muscle and is simply a biomarker of the aging process.

To address this question, we developed a model of partial mtDNA depletion to replicate that seen in aged human islets using the approach of targeted knock down of *TFAM* gene expression in MIN6 cells. TFAM is an important nuclear encoded mtDNA transcription factor found to play a key role in mtDNA transcription [23], [24], as well as mtDNA copy number [25] and maintenance [26]. Using our model we were able to replicate a similar degree of mtDNA copy number depletion that had been observed in the human islets with aging. Under these conditions, we found that partial mtDNA depletion significantly impaired mitochondrial gene transcription and translation, as well as mitochondrial oxidative respiration and ultimately, glucose-stimulated insulin secretion. The age-related decline in mtDNA copy number observed in human islets could well contribute directly to the increased prevalence of type 2 diabetes with increasing age.

## Materials and Methods

### Cell Culture

MIN6 cells, a mouse pancreatic beta cell line established by Miyazaki *et al.* [27], were donated by Dr Susan Campbell and Dr Catherine Arden (Diabetes Research Group, Newcastle University, UK). All experiments were conducted between passages 23 and 31. Unless otherwise stated, cell culture reagents were manufactured by Gibco, and supplied by Life Technologies (Paisley, UK). MIN6 cells were cultured in DMEM supplemented with 25 mM D-glucose, L-glutamine, 15% filter sterilised FBS, 100 U/ml penicillin, 100 mg/ml streptomycin, and 0.0005% β-mercaptoethanol (Sigma, Dorset, UK). Cells were incubated at 37°C 5% CO_2_ in a humidified incubator and were passaged every 5–7 days.

### TFAM Silencing

*TFAM* was transcriptionally silenced in MIN6 cells using the Neon electroporation transfection system (Life Technologies, Paisely, UK). When cells reached ∼80% confluency, they were trypsinised and washed with PBS prior to cell counting using trypan blue exclusion. Cells were resuspended in Resuspension Buffer R, from the 10 µl Neon transfection kit (Life Technologies, Paisley, UK). Cells were transfected in solution at a density of 200,000 cells per well in 24 well plates using MIN6 growth medium without additional antibiotics. Two TFAM siRNA probes were used at a concentration of 20 µM per well: TFAM-193 (5′- CCUCGUCUAUCAGUCUUGUCUGUAU -3′; 3′- AUACAGACAA GACUGAUAGACGAGG -5′) and TFAM-429 (5′- UACAAAGAAGCUGUGAGCAAGU AUA -3′; 3′- UAUACUUGCUCACAGCUUCUUUGUA -5′), both were Stealth duplex siRNA synthesised by Life Technologies (Paisley, UK). A Scrambled medium GC content siRNA probe (Life Technologies, Paisley, UK) was used as a transfection control; cells that were electroporated (shocked) in the absence of any siRNA were used to control for siRNA toxicity. Transfected cells were incubated at 37°C 5% CO_2_ in a humidified incubator and were harvested 48 h and 72 h post transfection. Transfection efficiency was based on the degree of target *TFAM* gene knock down; transfections were only accepted for analysis if *TFAM* knock down was ≥80%.

### Real-Time PCR

Total DNA and RNA were extracted simultaneously using the AllPrep DNA/RNA mini extraction kit (Qiagen, Crawley, UK), or separately using the GenElute Mammalian Total RNA miniprep kit (Sigma, Dorset, UK) or the DNeasy Blood & Tissue DNA extraction kit (Qiagen, Crawley, UK). Data presented in this manuscript utilise DNA extracted using both the DNeasy and AllPrep kits, but despite a slightly reduced DNA yield using the AllPrep kit, the quality of extracted DNA was the same for both kits. RNA was quantified using the Agilent 2100 BioAnalyzer (Agilent Technologies UK Limited) and 150 ng reverse transcribed using the First Strand cDNA Synthesis Kit (Life Technologies, Paisley, UK). Messenger RNA for the *TFAM*, *COX1* and *Ins1* genes were detected using TaqMan hydrolysis probes obtained from Applied Biosystems (Life Technologies, Paisley, UK), and normalised to the reference gene β2-microglobulin (*B2M*). DNA was used to determine mtDNA copy number, as described below. Real-time PCR was conducted using the Roche LightCycler 480 thermo cycler (Roche Diagnostics Ltd) and PCR products were quantified fluorometrically using the LightCycler 480 Master I (Roche, Welwyn Garden City, UK) kit and TaqMan probes for RNA or the LightCycler 480 SYBR Green I Master (Roche, Welwyn Garden City, UK) kit for DNA. Quantification of gene expression was performed using the Delta Ct (ΔCt) method [28].

### mtDNA Copy Number Assay

mtDNA levels were measured by relative real-time PCR, calculating the ratio of the mtDNA encoded target gene *ND5* [29] to the nuclear DNA encoded reference gene *GAPDH*. DNA was extracted as described above, quantified using the NanoDrop ND-1000 Spectrophotometer (Labtech International Ltd) and 50 ng was amplified per PCR reaction using 300 nM ND5 primers (forward: 5′- CTGGCAGACGAACAAGAC -3′; reverse: 5′- GAGGCTTCCGATTACTAGG -3′) or 500 nM GAPDH primers (forward: 5′- CAATGTGTCCGTCGTGGATCT -3′; reverse: 5′- GTCCTCAGTGTAGCCCAAGAT -3′). Each reaction was optimized and confirmed linear over an appropriate concentration range (S1 Fig.). Gene quantification was performed by ΔCt [28] (S1 Table). The relative gene expression ratio obtained was then multiplied by two on account of *GAPDH* being diploid.

### Measurement of Mitochondrial Respiration

Mitochondrial respiration was measured in MIN6 cells 72 h after transfection using the Seahorse XF24 Analyzer (Seahorse Biosciences). Cells were transfected at a density of 200,000 per well as described above and seeded in a 24 well plate. Media was replaced with basic media containing FBS (3%), pyruvate (10 mM), L-glutamine (2 mM) and glucose (25 mM) and cells incubated in a CO_2_-free environment for 1 h prior to the experiment. Oxygen consumption rates (OCR) were measured in live cells in the absence and then presence of various compounds inhibiting specific mitochondrial complexes in order to assess mitochondrial activity. Oligomycin (1 µg/ml) was injected to inhibit Complex V (ATP Synthase), followed by sequential addition of carbonyl cyanide p-trifluoromethoxy-phenylhydrazone (FCCP) (2 µM and 3.5 µM) to uncouple respiration and promote maximal respiration, and finally antimycin A (2.5 µM) was injected to inhibit Complex III (Ubiquinol-Cytochrome *c* Reductase). Basal respiration was measured as the area under the curve prior to injection of oligomycin; maximal respiration was measured as the area under the curve following the first FCCP injection and prior to the antimycin injection. ATP synthesis by oxidative phosphorylation was calculated by multiplying the ATP turnover by 2.3 as described previously [30]–[32]; where ATP turnover was (basal OCR – non mitochondrial respiration) – (oligomycin-inhibited OCR – non mitochondrial respiration), and 2.3 was the established phosphate/oxygen ratio. Data were normalised to total protein content.

### Glucose-Stimulated Insulin Secretion (GSIS)

Method adapted from Ishihara *et al.* [33]. Briefly, cells were washed twice with Krebs-Hepes buffer (119 mM NaCl, 4.74 mM KCl, 2.54 mM CaCl_2_, 1.19 mM MgCl_2_, 1.19 mM KH_2_PO_4_, 25 mM NaHCO_3_, 10 mM HEPES, 0.5% BSA, pH 7.4) before pre-incubating with Krebs-Hepes buffer at 37°C for 30 min. Cells were then washed again before stimulating for 1 h at 37°C with either basal 3 mM or high 25 mM glucose, with or without 0.1 µM glibenclamide (Sigma, Dorset, UK) in Krebs-Hepes buffer. Cell medium was harvested and insulin secretion determined using the high range rat insulin ELISA kit (Mercodia AB, Upsala, Sweden). Insulin concentration of unknown samples was calculated using a standard curve of known insulin concentrations and was normalised to protein content.

### Insulin Content

After GSIS, cells were harvested in 100 µl distilled water and were sonicated for 10 sec. Insulin was liberated from cells by acid-ethanol extraction; 50 µl sample volume was added to 100 µl of 0.18 M HCl in 96% ethanol before incubating overnight at 4°C. Samples were then briefly vortexed and cell debris was removed following centrifugation at 1000 rpm for 5 min at 4°C. Insulin content was determined by insulin ELISA, after diluting samples at least 1∶100, and was normalised to whole cell protein content.

### Immunoblot Analysis

Whole cell protein lysates were harvested from MIN6 cells in ice cold protein extraction buffer (100 mM Tris-HCl, pH 7.4, 100 mM KCl, 1 mM EDTA, 25 mM Kf, 0.1% Triton X-100, 0.5 mM sodium orthovanadate, 1X protease inhibitor cocktail). Extracted proteins were quantified using the Coomassie Plus (Bradford) Protein Assay (Pierce, Thermo Fisher Scientific, Cramlington, UK) and 25 µg protein was resolved on a 12% SDS-polyacrylamide gel after denaturing at 37°C for 10 min. Resolved proteins were then transferred electrophoretically onto a nitrocellulose membrane before blocking with 5% Marvel solution (5% Marvel milk powder in TBS-T wash buffer; 65 mM Tris pH 7.4, 150 mM NaCl, 0.1% Tween-20) for 1 h at room temperature with agitation. Membranes were then incubated at 4°C overnight in the presence of either a mouse COX1 antibody diluted 1∶10,000 (MitoSciences, Abcam, Cambridge, UK; Catalogue No. MS404); a mouse SDH70 antibody diluted 1∶10,000 (MitoSciences, Abcam, Cambridge, UK; Catalogue No. MS204); or a mouse β-Actin antibody diluted 1∶10,000 (Sigma, Dorset, UK; Catalogue No. A5441). A goat anti-mouse IgG antibody conjugated to horse radish peroxidase (1∶2000) (Sigma, Dorset, UK; Catalogue No. A3673) was then added and the membrane incubated for 1 h at room temperature. Bound antibodies were detected by addition of an enhanced chemiluminescent solution (SuperSignal West Pico, Thermo Fisher Scientific, Cramlington, UK) and immunoreactive products were visualised following exposure to blue X-ray film (Thermo Fisher Scientific, Cramlington, UK) for less than 1 min. Protein bands were quantified using the GS-800 Calibrated Densitometer (BioRad Laboratories, Bath, UK) and the Quantity One 4.2.3 BioRad software. β-Actin was used as a loading control.

### Statistical Analysis

All statistical analyses were performed using GraphPad Prism version 5.01 (GraphPad Software, San Diego, California, USA). Data presented as means ± standard error of the mean (SEM), unless otherwise stated, with the number of experimental repeats provided in the figure legend. Significance was tested with one-way ANOVA followed by an unpaired t test. A probability (p) value <0.05 was considered statistically significant and <0.01 highly significant.

## Results

### TFAM transcriptional silencing causes partial mtDNA depletion

**Figure 1:**
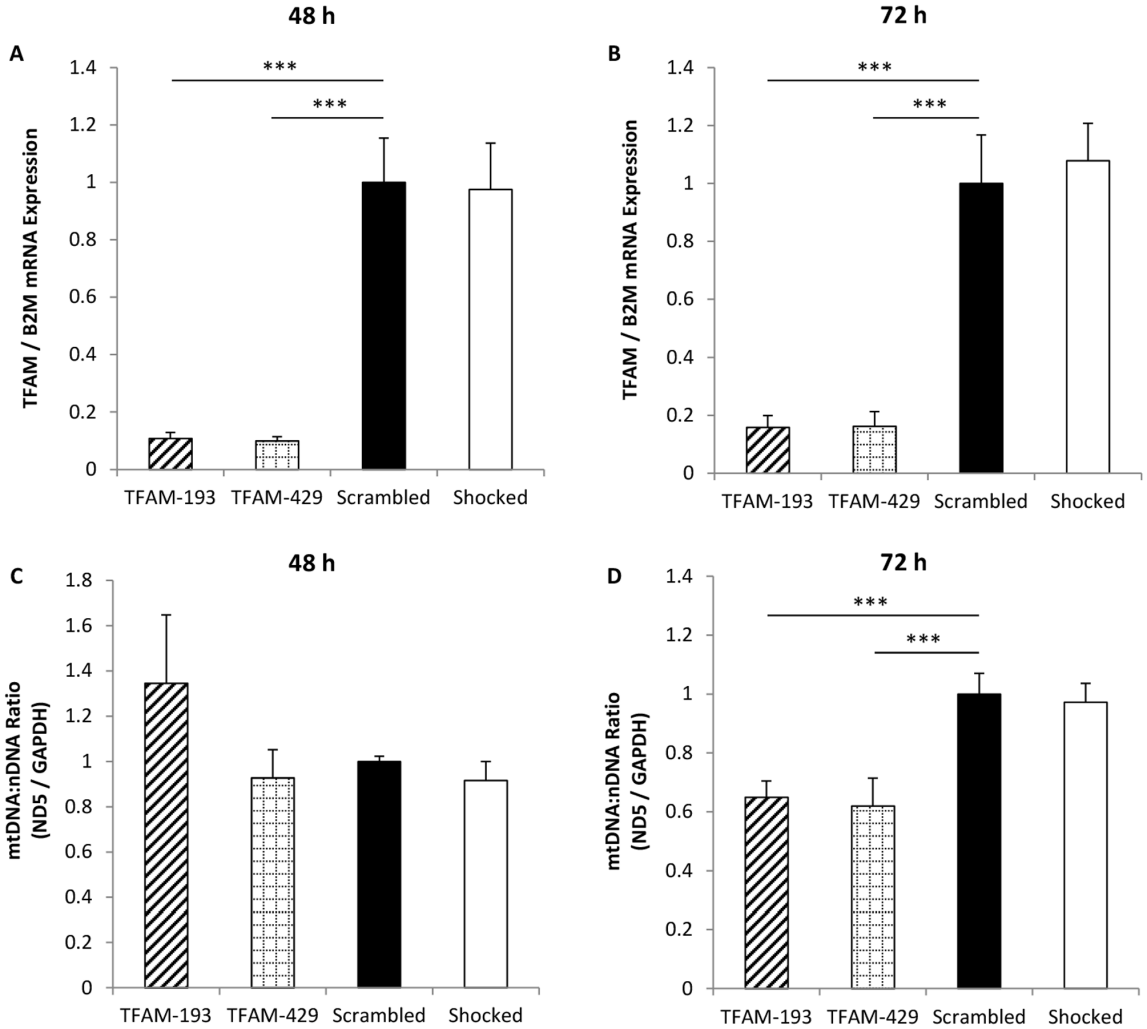
TFAM mRNA silencing induces mtDNA depletion 72 h post transfection. MIN6 cells were transfected with TFAM-193, TFAM-429 or Scrambled siRNA probes, or with no siRNA (Shocked). TFAM mRNA expression was quantified relative to reference gene *B2M* by real-time PCR at 48 h (A) and 72 h (B) post transfection. mtDNA depletion was also measured by real-time PCR, using mitochondrial encoded *ND5* relative to nuclear encoded *GAPDH* at 48 h (C) and 72 h (D) post transfection. All results normalised to Scrambled negative control. Experiment repeated once (C), twice (A) or 4 times (B, D) in triplicate. Data presented are means ± SEM (SD in (C)). * p<0.05, *** p<0.001.B2M, β2 Microglobulin; GAPDH, Glyceraldehyde-3-Phosphate Dehydrogenase; ND5, NADH Dehydrogenase 5; TFAM, Mitochondrial Transcription Factor A.

In order to partially deplete mtDNA levels, we used siRNA technology to transcriptionally silence the *TFAM* gene. TFAM was chosen as a target gene through its known roles in regulating mtDNA copy number [25], mtDNA transcription [23], [24], as well as mtDNA stability [26]. MIN6 cells were transfected using electroporation and two TFAM siRNA duplexes targeting nucleotides 193 and 429 of the TFAM mRNA molecule. Transfected cells were harvested at 48 and 72 h post transfection. After 48 h (Fig. 1A) and 72 h (Fig. 1B), TFAM mRNA levels were markedly decreased by>80% with both the TFAM-193 and TFAM-429 probes when compared with the Scrambled negative control (p = 0.0001). DNA was extracted and used to calculate mtDNA levels by quantifying mtDNA encoded target gene *ND5* relative to nuclear encoded reference gene *GAPDH*. Interestingly, after 48 h post transfection mtDNA levels seem unaffected by the *TFAM* transcriptional silencing (Fig. 1C). However, after 72 h post transfection, there was around a 40% reduction in mtDNA levels with both the TFAM-193 and TFAM-429 probes when compared with the Scrambled negative control (p<0.001) (Fig. 1D). We validated this mtDNA depletion using a second nuclear encoded reference gene, *CDKN2A* (cyclin-dependant kinase inhibitor 2A) (S2 Fig.), and also found that *TFAM* transcriptional silencing with both probes resulted in the same 40% reduction in mtDNA levels 72 h post transfection. Therefore, using both the TFAM-193 and TFAM-429 siRNA probes, we successfully silenced TFAM mRNA by>80% 72 h post transfection, and this resulted in a 40% decrease in mtDNA levels.

### Partial mtDNA depletion affects mitochondrial gene expression

**Figure 2:**
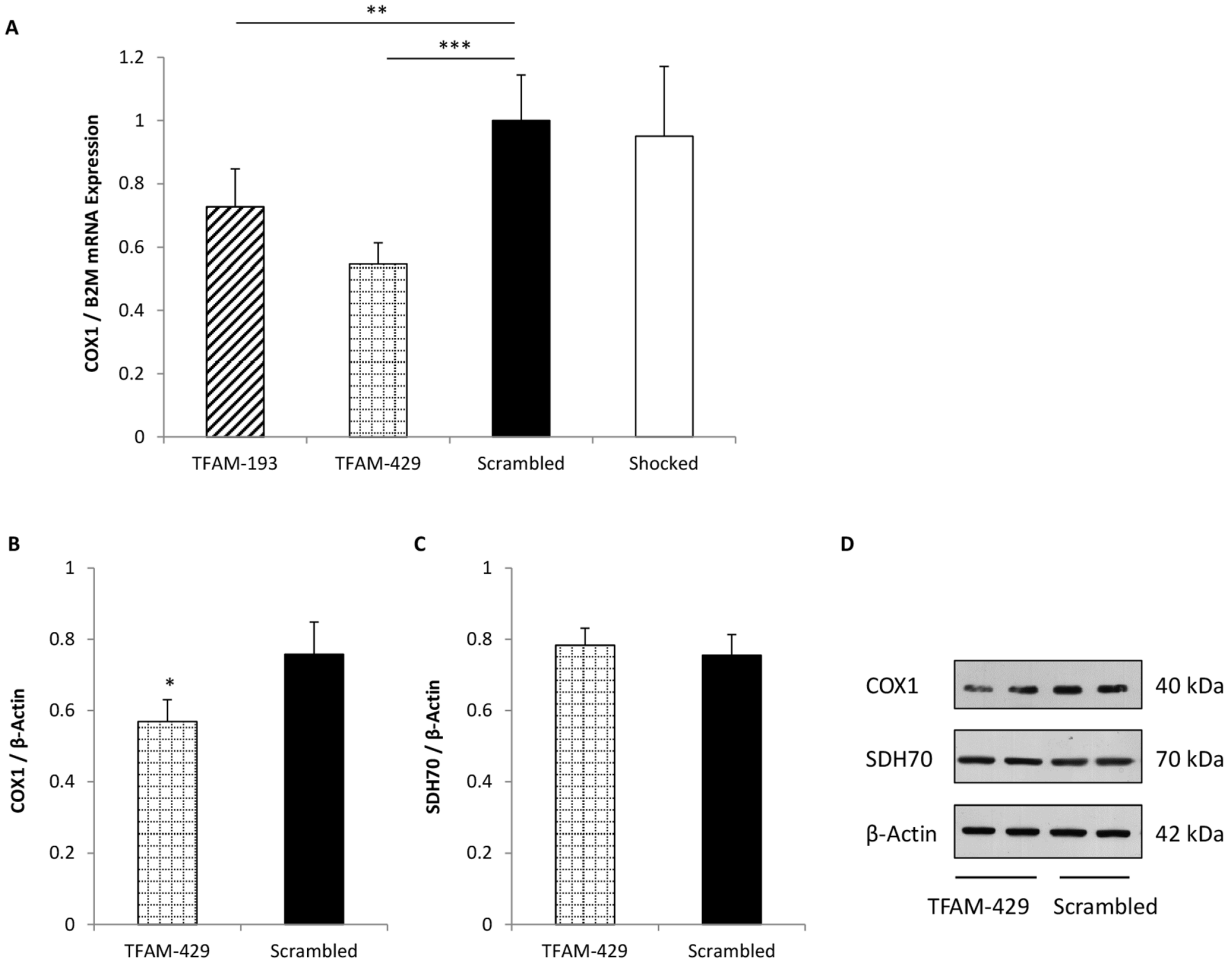
The effect of partial mtDNA depletion on mitochondrial gene transcription and protein translation. Mitochondrial DNA was depleted following *TFAM* silencing and COX1 mRNA expression was quantified relative to reference gene *B2M* 72 h post transfection (A). Protein was extracted 72 h post transfection and analysed by western blotting, probing for COX1, SDH70, and β-Actin proteins. Protein bands were quantified by densitometry, and optical density readings used to calculate the ratio of COX1 (B) and SDH70 (C) mitochondrial proteins relative to β-Actin loading control. A representative blot is shown in (D). Data in (A) are normalised to Scrambled control cells. Both experiments repeated 3 times, with each experimental repeat performed in triplicate (A) or duplicate (B, C). Data presented are means ± SEM. * p<0.05, ** p<0.01, *** p<0.001. COX1, Cytochrome *c* Oxidase 1; SDH70, Succinate Dehydrogenase 70 kDa subunit.

To investigate whether decreased mtDNA levels following *TFAM* gene silencing affected mitochondrial gene expression, we looked at transcription and translation of the mtDNA encoded gene *COX1*, cytochrome *c* oxidase 1. COX1 is one of the three mtDNA encoded subunits that comprise the catalytic holoenzyme of Complex IV, Cytochrome *c* Oxidase (COX), of the respiratory chain [26], [34]. As before, MIN6 cells were transfected and incubated for 72 h after which, either whole cell RNA or protein was harvested. COX1 mRNA expression relative to reference gene *B2M* was determined by real-time PCR. As shown in Fig. 2A, COX1 mRNA levels were significantly decreased by 24% (p<0.01) and 33% (p<0.001) in cells transfected with the TFAM-193 and TFAM-429 probes respectively, compared with the Scrambled control. Because the TFAM-429 siRNA probe produced the greatest decrease in COX1 mRNA levels, we used this probe to examine the effect of mtDNA depletion on COX1 protein levels. We found that the decrease in COX1 mRNA led to a 25% decrease in COX1 protein expression (p = 0.034) (Fig. 2B and 2D). We also investigated the nuclear encoded protein SDH70, a 70 kDa component of Complex II, succinate dehydrogenase, of the respiratory chain. Unlike the other respiratory complexes, succinate dehydrogenase is entirely nuclear encoded, and should not be affected by mtDNA depletion. This was confirmed as SDH70 protein levels were unaffected by *TFAM* silencing-induced mtDNA depletion (Fig. 2C and 2D).

### Partial mtDNA depletion impairs mitochondrial function

**Figure 3:**
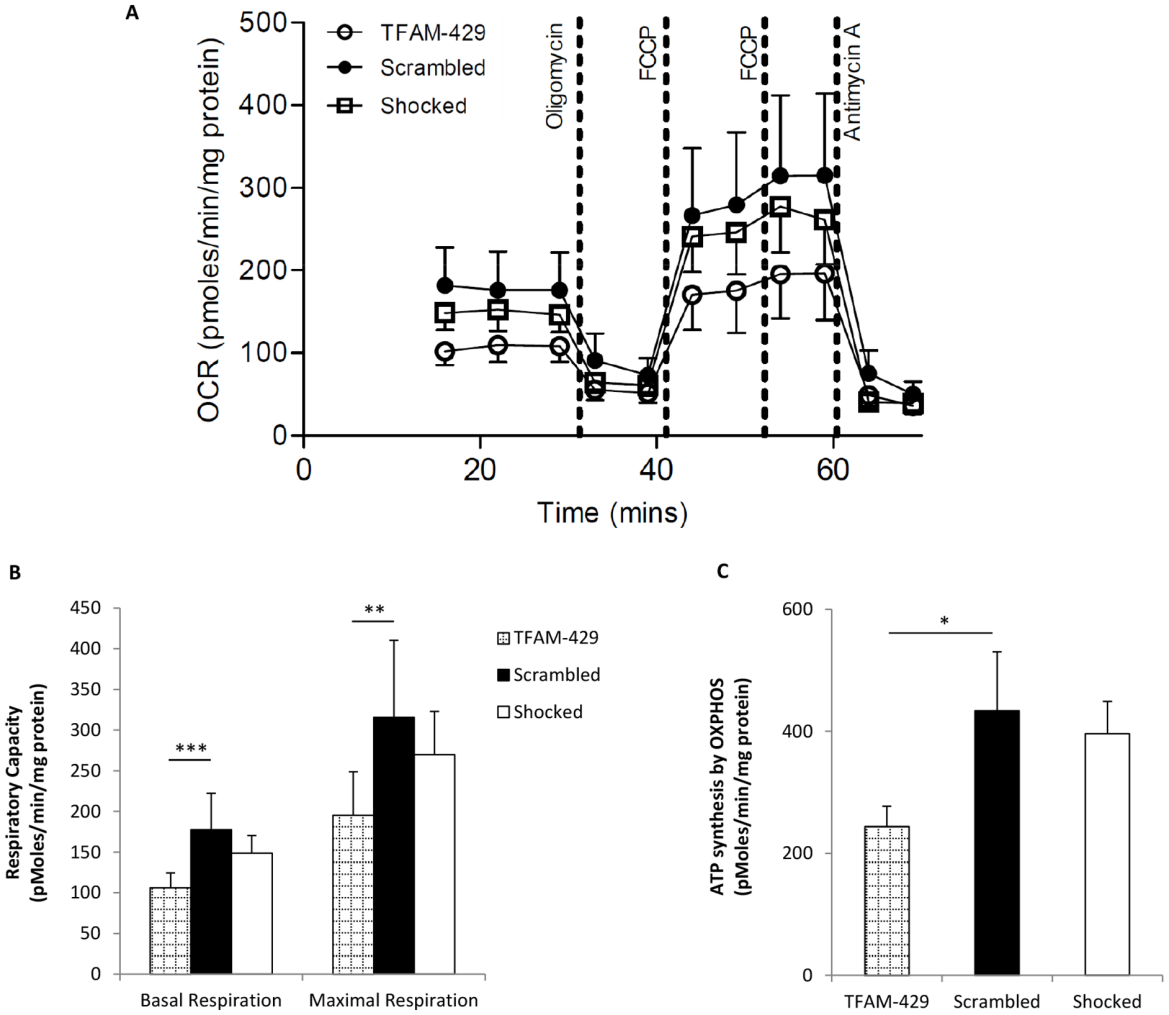
The effect of partial mtDNA depletion on mitochondrial function. Cells were harvested 72 h post transfection and oxygen consumption rate (OCR) was measured using the Seahorse XF24 Analyzer. OCR in TFAM-429 cells (n = 8) was severely impaired compared to that of Scrambled (n = 8) and Shocked (n = 8) control cells (A). Mitochondrial activity was measured following injection of oligomycin, an inhibitor of Complex V (ATP Synthase), followed by two sequential injections of FCCP to uncouple respiration and induce maximal respiration, and finally antimycin, an inhibitor of Complex III (Ubiquinol-Cytochrome *c* Reductase) preventing electron transfer and subsequently abolishing the proton gradient required for ATP synthesis. Basal and maximal respiratory capacity (B) and ATP synthesis by oxidative phosphorylation (OXPHOS) (C) were calculated as described previously [30]–[32]. Data were normalised to protein concentration and are presented means ± SEM. * p<0.5, ** p<0.001, *** p<0.0001.

The Seahorse XF24 Analyzer was used to measure mitochondrial respiratory capacity in transfected cells (Fig. 3A). Both basal and maximal respiration were significantly impaired in siRNA TFAM transfected cells compared to scrambled control (p = 0.0005 and p = 0.005, respectively) (Fig. 3B). Basal respiratory capacity is represented by the area under the curve prior to oligomycin injection, whereas maximal respiratory capacity is defined as the area under the curve between the first FCCP injection and the antimycin injection (Fig. 3A) and is presented graphically in Fig. 3B. Consistent with a reduced basal respiration, siRNA TFAM transfected cells also showed significantly reduced ATP synthesis due to oxidative phosphorylation (p<0.05) (Fig. 3C).

### Partial mtDNA depletion impairs glucose-stimulated insulin secretion

**Figure 4:**
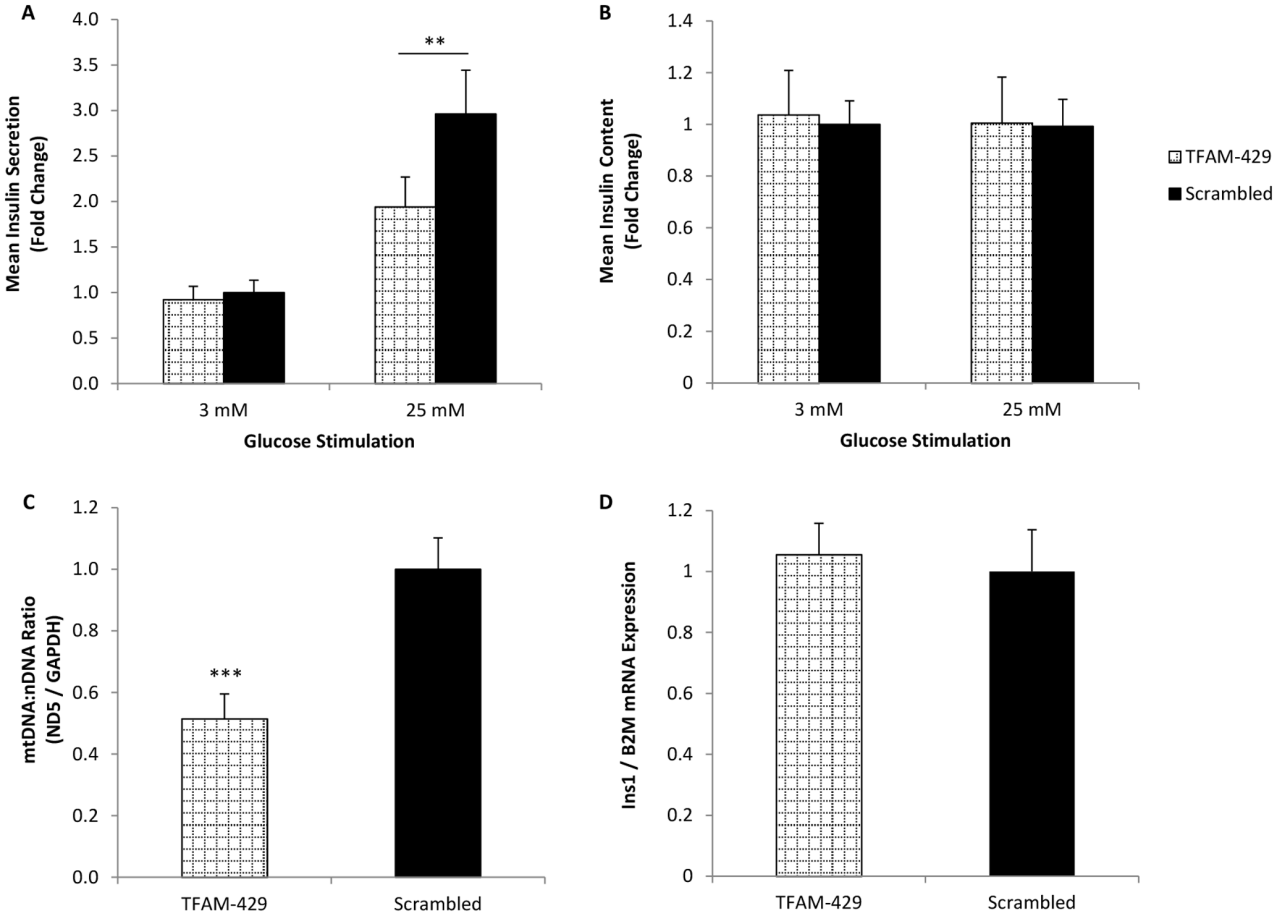
The effect of partial mtDNA depletion on glucose-stimulated insulin secretion. Seventy two hours post transfection cells were stimulated with basal (3 mM) or high (25 mM) glucose concentrations. Insulin secretion (A) and insulin content (B) were determined by insulin ELISA and normalised to protein content. Data normalised to 3 mM glucose stimulated Scrambled control cells. mtDNA levels (C) and *Ins1* insulin gene expression (D) were quantified and normalised to the Scrambled control. Data shown are from 9 (A) or 3 (B, C, D) separate experiments, each performed in triplicate. Data presented are means ± SEM. ** p≤0.01, *** p<0.001.

Having demonstrated that partial mtDNA depletion following *TFAM* gene silencing directly affects mitochondrial gene expression and mitochondrial function, we were keen to establish whether this in turn affects glucose-stimulated insulin secretion. Seventy-two hours post transfection, MIN6 cells were stimulated with either basal 3 mM glucose or high 25 mM glucose for a period of 1 h. As shown in Fig. 4A, both TFAM-429 transfected cells and Scrambled control cells both responded to the higher 25 mM glucose stimulation by secreting significantly more insulin, when compared with basal 3 mM stimulation (p = 0.0004 and p<0.0001 respectively). What is interesting however, is that insulin secretion was significantly impaired in *TFAM* silencing-induced mtDNA depleted cells following 25 mM glucose stimulation (p = 0.003). The impaired insulin secretion observed in mtDNA depleted cells was not a consequence of altered insulin content following TFAM knock down (Fig. 4B). We confirmed that partial mtDNA depletion was achieved as before under these conditions (Fig. 4C). Fig. 4D shows that mtDNA depletion did not alter *Ins1* insulin gene expression.

### Glibenclamide restores the impaired insulin secretion seen following mtDNA depletion

**Figure 5:**
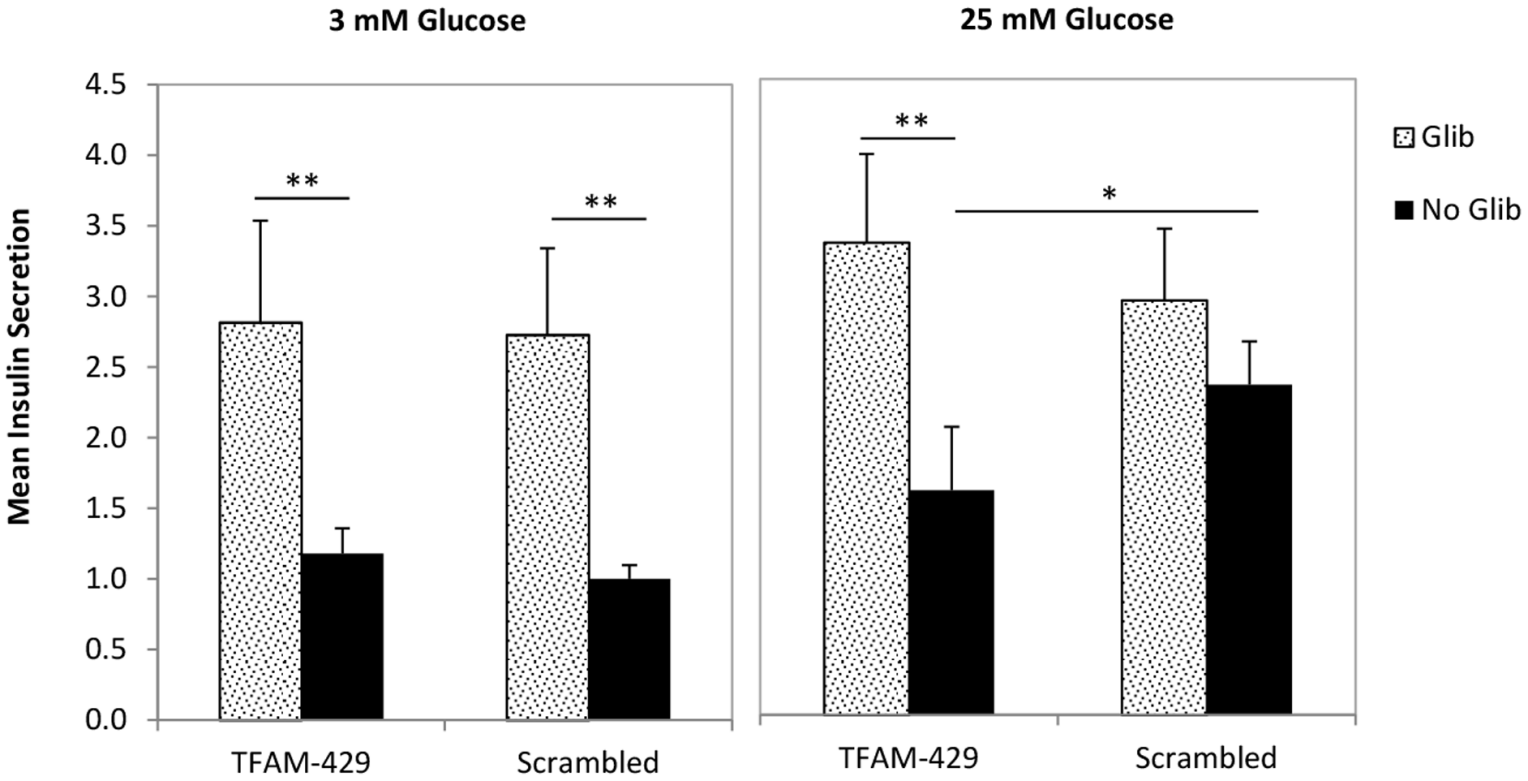
The effect of glibenclamide on insulin secretion following *TFAM* silencing-induced mtDNA depletion. Seventy two hours post transfection cells were stimulated with 3 mM or 25 mM glucose, supplemented with or without 0.1 µM glibenclamide. Insulin secretion was determined by insulin ELISA and normalised to whole cell protein content. Data shown are from 4 separated experiments performed in triplicate, and are normalised to Scrambled negative control cells stimulated with 3 mM glucose without glibenclamide. Data presented are means ± SEM. * p<0.05, ** p<0.01.

Glibenclamide is a second generation sulphonylurea. By targeting the sulphonylurea 1 (SUR1) protein on the beta cell K^+^ channel, it promotes channel closure and subsequent pancreatic beta cell membrane depolarisation and insulin secretion [35]. Consequently, glibenclamide bypasses the step of ATP generation by the mitochondria and is therefore a useful drug to help determine whether or not a deficit in insulin secretion is mitochondrial in origin. To see whether glibenclamide treatment had any effect on insulin secretion in mtDNA depleted cells, transfected cells were stimulated with either 3 mM or 25 mM glucose for 1 h, with or without 0.1 µM glibenclamide. The siRNA data corroborate what we have seen previously, that insulin secretion (in the absence of glibenclamide) is impaired in TFAM-429 cells after 25 mM glucose stimulation compared with Scrambled control cells (p<0.05) (Fig. 5). At 3 mM glucose, 0.1 µM glibenclamide increased insulin secretion to a comparable degree in TFAM-429 cells and Scrambled control cells (Fig. 5) compared to no glibenclamide (both p<0.01). At 25 mM glucose, glibenclamide fully restored insulin secretion to normal in the TFAM-429 cells compared with Scrambled control cells, indicating that the impact of mtDNA depletion is focused at the step of mitochondrial respiration.

### Discussion

The primary objective of our work was to generate a model of partial mtDNA depletion by genetically silencing mitochondrial transcription factor A, TFAM. Indeed, tissue specific [19], [36], [37] and global knockdown [38] of the *TFAM* gene in mouse animal models has proven an effective means of depleting mtDNA levels. After transcriptionally silencing the *TFAM* gene in MIN6 cells by>80%, we achieved a 40% reduction in mtDNA levels, which was comparable to the degree of mtDNA depletion seen in aged human islets [14].

COX1, cytochrome *c* oxidase subunit 1, is one of the three mtDNA encoded subunits of Cytochrome *c* Oxidase (COX, Complex IV), and is essential during the assembly of the Cytochrome *c* Oxidase complex [26], [34]. Partial mtDNA depletion significantly decreased COX1 mRNA and protein expression by 33% and 25%, respectively. Our finding that mtDNA depletion results in decreased mtDNA transcription and protein translation has also been shown in previous studies in rodent clonal beta cells [20], [21], [39] and rodent pancreatic islets [40]. We have also demonstrated that partial mtDNA depletion had a direct impact on mitochondrial function. We found a significant decrease in basal and maximal respiratory capacity, as well as a significant reduction in ATP production by oxidative phosphorylation in *TFAM*-silenced cells compared to control cells. However, these results were founded on the assumption that the phosphate/oxygen ratio remained constant, which may not have been the case under conditions of mtDNA depletion [41].

Previous studies have observed decreased insulin secretion after severe (around 90%) depletion of mtDNA depleted pancreatic beta cells [18]–[21], [39]. However, this magnitude of severe mtDNA depletion does not accurately reflect the degree of age-related mtDNA depletion observed in human islets. In human pancreatic islets, it was noted that there was an average 50% decrease in mtDNA copy number in non-diabetic islet donors aged between 17 and 75 years [14]. In this study mtDNA levels were depleted relative to the control by an average of 40%, close to the degree of depletion observed in human islets. This level of mtDNA depletion resulted in a significant decrease in insulin secretion following 25 mM high glucose stimulation. This supports the idea that the age-related depletion in mtDNA has a direct impact on beta cell function and is not simply a biomarker of beta cell aging. Ihm *et al.* correlated the decline in glucose-stimulated insulin secretion and decreased ATP content to islet donor age [42]. Gauthier *et al.* observed partial mtDNA depletion to similar levels produced by ourselves following transcriptional silencing of the beta cell regulatory transcription factor Pdx1 [40]. By silencing *Pdx1* gene expression by>90%, the group found this depleted mtDNA levels by 40% via *TFAM* suppression, which resulted in impaired insulin secretion at high glucose stimulation only [40]. This model differs from our targeted knockdown of *TFAM* in that Pdx1 is a key transcription factor that regulates the expression of multiple genes involved in pancreatic function beyond the mitochondria [43].

Gene variants in other mitochondrial transcription factors, namely TFB1M, have been found to correlate with reduced insulin secretion, elevated postprandial glucose levels and increased risk of developing type 2 diabetes [44]. TFB1M actually functions as a methyltransferase as opposed to a transcription factor [45], [46], but this work serves to show that mutations in nuclear encoded mitochondrial genes can be diabetogenic.

Taking these data together it would suggest that partial mtDNA depletion to levels observed in aging has a direct and detrimental effect on pancreatic beta cell function. This seems to be different to the findings in other tissues. Specifically, COX deficiency as a marker of mitochondrial dysfunction was only seen when the degree of mtDNA depletion was ≥95% in human skeletal muscle [22]. This implies that the degree of mtDNA depletion required to impair mitochondrial dysfunction differs between tissues. The reason for this is not clear. It may be that the absolute amounts of mtDNA differ between cells types, so that those with comparatively lower levels are more susceptible to the effects of depletion. It may be that mechanisms to maintain mitochondrial function and ATP production in the face of mtDNA depletion differ between cells types. Whatever the reason, it appears that pancreatic islet cells are particularly sensitive to mtDNA depletion and altered mitochondrial function. This is in keeping with our earlier observation that comparatively low levels of mutated to wild type mtDNA were found in pancreatic islet cells in a patient with diabetes secondary to the A3243G mtDNA mutation [12]. Interestingly, a mouse model of marked peripheral insulin resistance was characterised by an age-related development of diabetes that was linked to a decrease in both pancreatic beta cell mtDNA content and mitochondrial function, and decreased glucose-stimulated insulin secretion [47].

What might be causing the reduced insulin secretion seen in *TFAM* silenced mtDNA depleted cells? We found no change in the *Ins1* insulin gene expression in *TFAM* silenced mtDNA depleted cells, so the defect does not appear to alter insulin gene transcription. This is supported by our finding that total insulin content remained relatively unchanged in *TFAM* silenced cells compared to control cells, after 3 mM and 25 mM glucose stimulation. Nonetheless insulin secretion, when expressed as a percentage of total insulin content, was still reduced in *TFAM*-transfected cells (S2 Table).

Glibenclamide is currently an effective treatment for type 2 diabetes patients with impaired insulin secretion [48], although its clinical application has been generally superseded by sulphonylureas with shorter half-lives. By targeting the sulphonylurea receptor (SUR1) protein of the ATP-gated K^+^ channel situated on the beta cell plasma membrane, it promotes membrane depolarisation, and subsequent insulin secretion following Ca^2+^-stimulated exocytosis [35] and therefore, acts downstream to the step of mitochondrial respiration and ATP production. We found that insulin secretion is fully restored to normal in the mtDNA depleted cells following addition of glibenclamide, so confirming that the key impact of mtDNA depletion is at the level of mitochondrial respiration.

A potential limitation of our work could be that the effects of *TFAM* gene silencing on mtDNA are transient and so, future studies into the effect of chronic partial mtDNA depletion might more accurately reflect mtDNA depletion in a clinical setting. Silva *et al.* produced pancreatic beta cell specific TFAM knock down and severe mtDNA depletion in mice [19]. The mice initially developed diabetes due to impaired glucose-stimulated insulin secretion just as we saw in our model, but as the animals aged there was a concurrent decrease in beta cell mass that sustained the diabetes phenotype. We found no evidence of decreased cell mass in our cell line model, based upon comparable DNA, RNA and protein levels between TFAM knock down cells and the Scrambled control cells. This does not accurately reflect what is seen *in vivo* or clinically in type 2 diabetes patients and so, our studies would need to be repeated using mouse models to further elucidate the effects of partial mtDNA depletion on diabetes pathogenesis. Finally, we have demonstrated that the partial mtDNA depletion produced in our MIN6 cell line model contributes to reduced mitochondrial function in terms of oxygen consumption; however, further characterisation of mitochondrial dysfunction following evaluation of COX activity would be beneficial for future studies.

In conclusion, we found that mtDNA depletion in MIN6 cells to levels seen in human islets with aging has a direct effect on insulin secretion when depleting mtDNA via *TFAM* gene silencing. This effect on insulin secretion may be due to a defective electron transport chain following a decrease in the mtDNA encoded components, resulting in impaired mitochondrial respiration and ATP production. We found that the impaired insulin secretion was restored following treatment with the insulin secretagogue glibenclamide, suggesting that the deficit in insulin secretion occurs upstream of the K^+^ channel closure and is mitochondrial in origin. Strategies to slow or even prevent islet mtDNA depletion in man could help to preserve insulin secretion and delay the development of type 2 diabetes.

## Supporting Information

S1 Fig **Optimisation of mtDNA Copy Number Assay.** Real-time PCR reactions for GAPDH and ND5 primers were first optimised using a linear standard curve: 50 ng DNA was serially diluted 1∶5, before amplification with GAPDH, ND5 or CDKN2A primers. Standard curves were confirmed linear over an appropriate concentration range. Reaction efficiencies were 92.61% (GAPDH), 98.87% (ND5) and 95.07% (CDKN2A). Reaction specificity was tested by dissociation curve as well as agarose gel electrophoresis of PCR products.
(DOCX)

S2 Fig **Validation of mtDNA assay using a second nuclear encoded gene.** The mtDNA copy number assay was validated by quantifying *ND5* relative to a second nuclear encoded reference gene, *CDKN2A*, cyclin-dependent kinase inhibitor 2A (QuantiTect Assay ID Mm_Cdkn2a_va.1_SG; Qiagen, Crawley, UK). Cells were harvested 72 hrs post transfection and mtDNA depletion determined by relative real-time PCR and normalisation to Scrambled control cells. Experiment repeated twice in triplicate, error bars represent SEM. * p<0.05.
(DOCX)

S1 Table **Mitochondrial encoded ND5 expression relative to nuclear encoded GAPDH gene content as determined by differences in Ct values.** mtDNA copy number was determined as the ratio of target mtDNA gene *ND5* relative to reference nDNA gene *GAPDH* using the Delta-Ct (ΔCt) method [28]. The cycle threshold, or Ct, was determined by real-time PCR and ΔCt was calculated as the difference in Ct values between the target gene and the reference gene. Change in gene expression was calculated by 2(2^−ΔCt^).
(DOCX)

S2 Table **Glucose-stimulated insulin secretion normalised to total insulin content.** The table above represents the data values used to construct Fig. 4A and 4B. Percentage insulin secretion was calculated by normalising insulin secreted by total insulin content.
(DOCX)
