# Supplementary material for: Age-Related Mitochondrial DNA Depletion and the Impact on Pancreatic Beta Cell Function
Source: PLoS One. 2014 Dec 22;9(12):e115433. doi: 10.1371/journal.pone.0115433 (PMC4274008; doi:10.1371/journal.pone.0115433)
Supplement: S1 Fig — Optimisation of mtDNA Copy Number Assay. Real-time PCR reactions for GAPDH and ND5 primers were first optimised using a linear standard curve: 50 ng DNA was serially diluted 1∶5, before amplification with GAPDH, ND5 or CDKN2A primers. Standard curves were confirmed linear over an appropriate concentration range. Reaction efficiencies were 92.61% (GAPDH), 98.87% (ND5) and 95.07% (CDKN2A). Reaction specificity was tested by dissociation curve as well as agarose gel electrophoresis of PCR products. (DOCX) [file pone.0115433.s001.docx]

Figure S1. Optimisation of mtDNA Copy Number Assay. Real-time PCR reactions for GAPDH and ND5 primers were first optimised using a linear standard curve: 50 ng DNA was serially diluted 1:5, before amplification with GAPDH, ND5 or CDKN2A primers. Standard curves were confirmed linear over an appropriate concentration range. Reaction efficiencies were 92.61% (GAPDH), 98.87% (ND5) and 95.07% (CDKN2A). Reaction specificity was tested by dissociation curve as well as agarose gel electrophoresis of PCR products.
