# Supplementary material for: Age-Related Mitochondrial DNA Depletion and the Impact on Pancreatic Beta Cell Function
Source: PLoS One. 2014 Dec 22;9(12):e115433. doi: 10.1371/journal.pone.0115433 (PMC4274008; doi:10.1371/journal.pone.0115433)
Supplement: S2 Fig — Validation of mtDNA assay using a second nuclear encoded gene. The mtDNA copy number assay was validated by quantifying ND5 relative to a second nuclear encoded reference gene, CDKN2A, cyclin-dependent kinase inhibitor 2A (QuantiTect Assay ID Mm_Cdkn2a_va.1_SG; Qiagen, Crawley, UK). Cells were harvested 72 hrs post transfection and mtDNA depletion determined by relative real-time PCR and normalisation to Scrambled control cells. Experiment repeated twice in triplicate, error bars represent SEM. * p<0.05. (DOCX) [file pone.0115433.s002.docx]

Figure S2. Validation of mtDNA assay using a second nuclear encoded gene. The mtDNA copy number assay was validated by quantifying *ND5* relative to a second nuclear encoded reference gene, *CDKN2A*, cyclin-dependent kinase inhibitor 2A (QuantiTect Assay ID Mm_Cdkn2a_va.1_SG; Qiagen, Crawley, UK). Cells were harvested 72 hrs post transfection and mtDNA depletion determined by relative real-time PCR and normalisation to Scrambled control cells. Experiment repeated twice in triplicate, error bars represent SEM. * p<0.05

*

*
