# Supplementary material for: Age-Related Mitochondrial DNA Depletion and the Impact on Pancreatic Beta Cell Function
Source: PLoS One. 2014 Dec 22;9(12):e115433. doi: 10.1371/journal.pone.0115433 (PMC4274008; doi:10.1371/journal.pone.0115433)
Supplement: S1 Table — Mitochondrial encoded ND5 expression relative to nuclear encoded GAPDH gene content as determined by differences in Ct values. mtDNA copy number was determined as the ratio of target mtDNA gene ND5 relative to reference nDNA gene GAPDH using the Delta-Ct (ΔCt) method [28]. The cycle threshold, or Ct, was determined by real-time PCR and ΔCt was calculated as the difference in Ct values between the target gene and the reference gene. Change in gene expression was calculated by 2(2−ΔCt). (DOCX) [file pone.0115433.s003.docx]

Table S1. Mitochondrial encoded ND5 expression relative to nuclear encoded GAPDH gene content as determined by differences in Ct values. mtDNA copy number was determined as the ratio of target mtDNA gene *ND5* relative to reference nDNA gene *GAPDH* using the Delta-Ct (ΔCt) method [28]. The cycle threshold, or Ct, was determined by real-time PCR and ΔCt was calculated as the difference in Ct values between the target gene and the reference gene. Change in gene expression was calculated by 2(2^-ΔCt^ ).

|  | **mtDNA** | **nDNA** | **^a^ ΔCt** | **^b^ 2^-ΔCt^** | **^c^ 2(2^-ΔCt^ )** | **Normalised** |
| --- | --- | --- | --- | --- | --- | --- |
|  | **ND5 Ct**  **(mean ± SEM)** | **GAPDH Ct (mean ± SEM)** |  |  |  |  |
| **TFAM-193** | 20.29 ± 0.55 | 19.61 ± 0.80 | 0.674 | 0.627 | 1.254 | 0.64 |
| **TFAM-429** | 20.27 ± 0.69 | 19.53 ± 0.88 | 0.741 | 0.598 | 1.197 | 0.61 |
| **Scrambled** | 19.87 ± 0.62 | 19.84 ± 0.81 | 0.026 | 0.982 | 1.965 | 1.0 |
| **Shocked** | 19.78 ± 0.64 | 19.81 ± 0.77 | 0.030 | 1.021 | 2.042 | 1.02 |

^a^ ΔCt = Ct_ND5_ – Ct_GAPDH_

^b^ Accounts for the doubling of DNA after each PCR cycle, i.e. a reaction efficiency of 2

^c^ Multiply by 2 on account of GAPDH being diploid
