# Supplementary material for: Age-Related Mitochondrial DNA Depletion and the Impact on Pancreatic Beta Cell Function
Source: PLoS One. 2014 Dec 22;9(12):e115433. doi: 10.1371/journal.pone.0115433 (PMC4274008; doi:10.1371/journal.pone.0115433)
Supplement: S2 Table — Glucose-stimulated insulin secretion normalised to total insulin content. The table above represents the data values used to construct Fig. 4A and 4B. Percentage insulin secretion was calculated by normalising insulin secreted by total insulin content. (DOCX) [file pone.0115433.s004.docx]

**Table S2. Glucose-stimulated insulin secretion normalised to total insulin content.** The table above represents the data values used to construct Fig. 4A and 4B. Percentage insulin secretion was calculated by normalising insulin secreted by total insulin content.

|  | **Glucose Stimulation** | **Insulin secretion (pg/µg)**  **(mean ± SEM)** | **Insulin content (pg/µg)**  **(mean ± SEM)** | **Percentage insulin secretion (secretion/content)** |
| --- | --- | --- | --- | --- |
| **TFAM-429** | 3 mM | 352.27 ± 56.32 | 26672 ± 4394.94 | 1.32% |
|  | 25 mM | 741.53 ± 125.02 | 25870.79 ± 4548.86 | 2.87% |
| **Scrambled** | 3 mM | 382.00 ± 51.86 | 25715.19 ± 2325.90 | 1.49% |
|  | 25 mM | 1131.33 ± 184.02 | 25511.71 ± 2674.12 | 4.43% |
